# Erosive Potential of Commercially Available Cannabis Beverages

**DOI:** 10.1002/cre2.70378

**Published:** 2026-05-15

**Authors:** Budsaraporn Boonsuth, Lawan Boonprakong, Ajjima Chansaenroj, Sureeporn Tantisak, Thawanrat Singthong, Vorapat Trachoo, Sant Ananchanachai, Naruporn Monmaturapoj, Supreda Suphanantachat Srithanyarat, Dusit Nantanapiboon, Junji Tagami, Thanaphum Osathanon

**Affiliations:** ^1^ Oral Biology Research Center, Faculty of Dentistry Chulalongkorn University Bangkok Thailand; ^2^ Office of Research and Innovation, Faculty of Dentistry Chulalongkorn University Bangkok Thailand; ^3^ Department of Anatomy, Center of Excellence for Dental Stem Cell Biology, Faculty of Dentistry Chulalongkorn University Bangkok Thailand; ^4^ Department of Animal Husbandry, Faculty of Veterinary Science Chulalongkorn University Bangkok Thailand; ^5^ Dental Materials Research and Development Center, Faculty of Dentistry Chulalongkorn University Bangkok Thailand; ^6^ Department of Oral and Maxillofacial Surgery, Faculty of Dentistry Chulalongkorn University Bangkok Thailand; ^7^ CU Dent Innovation, Inc Bangkok Thailand; ^8^ National Metal and Materials Technology Center Khlong Luang Pathum Thani Thailand; ^9^ Center of Excellence for Periodontology and Dental Implant, and Department of Periodontology, Faculty of Dentistry Chulalongkorn University Bangkok Thailand; ^10^ Department of Operative Dentistry, Faculty of Dentistry Chulalongkorn University Bangkok Thailand; ^11^ Clinic of General, Special Care, and Geriatric Dentistry, Center for Dental Medicine University of Zurich Zurich Switzerland; ^12^ Center of Excellence for Dental Stem Cell Biology, and Center of Excellence and Innovation for Oral Health and Healthy Longevity Faculty of Dentistry, Chulalongkorn University Bangkok Thailand; ^13^ Institution of Science Tokyo Tokyo Japan

**Keywords:** beverages, dental erosion, fluoride, hardness, surface properties

## Abstract

**Objective:**

To evaluate the erosive potential of commercially available cannabis beverages by assessing fluoride content and pH, as well as changes in microhardness and surface roughness.

**Materials and Methods:**

Sixteen cannabis‐infused beverages were analyzed, with distilled water used as the control. Fluoride concentration and pH were measured using a fluoride ion‐selective electrode and a calibrated pH meter. Eighty‐five synthetic enamel‐like hydroxyapatite blocks (5 mm diameter × 5 mm thickness) were prepared and allocated into 16 experimental groups and 1 control group (*n* = 5/group). Specimens were immersed in 5 mL of each beverage for 30 min at 37°C. Knoop microhardness (KHN) and surface roughness (Ra) were measured before and after immersion. The percentage of surface hardness loss (%SHL) and percentage change in surface roughness (%Raalt) were calculated. Statistical analysis was performed using one‐way ANOVA followed by Tukey's post hoc test (*p* < 0.05).

**Results:**

Most beverages exhibited acidic pH values, with 12 samples showing pH < 4. Fluoride concentrations ranged from 0.0268 to 1.8233 mg/L, with most samples containing levels insufficient to counteract erosive effects, although one beverage exceeded recommended fluoride limits. Beverages with lower pH were associated with significant reductions in microhardness and increases in surface roughness (*p* < 0.05). The percentage of surface hardness loss (%SHL) ranged from approximately 27% to 74% among acidic beverages, with the highest values observed in the most acidic formulations. In contrast, beverages with near‐neutral pH showed minimal changes, with %SHL below 10%. Similarly, %Raalt ranged from less than 10% in low‐erosive groups to over 900% in highly acidic beverages.

**Conclusion:**

Cannabis beverages with low pH exhibited significant erosive potential, as evidenced by decreased microhardness and increased surface roughness on synthetic enamel‐like hydroxyapatite substrates. Beverage acidity and formulation were key determinants of these effects.

## 1 Introduction

Cannabis beverages have gained notable attention in the wellness and functional beverage sector, driven by health‐conscious consumers seeking a modern, discreet, nonsmoking alternative to alcohol and carbonated soft drinks. A 2024 survey revealed that approximately 58% of participants were open to trying cannabidiol (CBD)‐infused drinks, and 53% would consider tetrahydrocannabinol (THC)‐infused versions, especially in familiar formats like juice and iced tea (Staples 2024). Additionally, research suggests cannabis may serve as a substitute for alcohol in some consumer populations, reinforcing its positioning as a viable alternative (Green 2024).

The main active ingredients in cannabis beverages are cannabinoids, particularly CBD and, in some cases, low levels of THC. CBD is a nonpsychoactive compound thought to provide potential therapeutic benefits, including anti‐inflammatory, antioxidant, anxiolytic, and analgesic properties (Miller et al. 2022; Atalay et al. 2019). Conversely, THC is the primary psychoactive compound responsible for mood and perception changes. Despite the growing popularity of cannabis beverages, several health concerns remain. Previous research had identified several limitations of oral CBD use, including inconsistent and inaccurate dosing, highly variable absorption (i.e., low and unpredictable bioavailability), and possible side effects such as drowsiness, gastrointestinal discomfort, or unintended interactions with other medications (Souza et al. 2022).

Beverages such as soft drinks, energy drinks, and fruit juices are well‐documented to cause dental erosion (Kumar et al. 2024). Dental erosion is defined as the irreversible loss of dental hard tissue caused by chemical processes not involving bacterial action. The mechanism underlying these effects involves both chemical and microbiological pathways (Lussi et al. 2023). Acidic ingredients, such as citric, phosphoric, and carbonic acids, lower the pH at the tooth surface, causing demineralization of hydroxyapatite crystals and progressive enamel erosion (Inchingolo et al. 2023). When these beverages also contain fermentable sugars, *Streptococcus mutans* and *Lactobacillus* spp. metabolize the sugars to produce organic acids, which further enhance demineralization and promote caries development (Wen et al. 2022). These detrimental effects were primarily associated with low pH and the acidic nature of such beverages, as supported by previous studies demonstrating reductions in enamel microhardness and increases in surface roughness (Barac et al. 2015; Trivedi et al. 2015).

Commercial cannabis beverages commonly contain acidic additives, fruit extracts, carbonated bases, and various sweeteners. However, their erosive potential and fluoride content remain poorly documented. Previous studies have reported that several CBD‐fortified beverages exhibit low pH values (≤ 4), comparable to conventional soft drinks, suggesting a potential risk for enamel demineralization and surface softening (Staples 2024). In addition, patent documentation indicates that cannabis beverages are often formulated within an acidic pH range of 4.0–5.0 using citric acid or similar acidulants (Green 2024), which may increase the risk of enamel erosion, as demineralization can occur below the critical pH of approximately 5.5.

Although cannabis beverages are widely marketed as safe and are readily available, their erosive potential remains unclear. Therefore, this study aimed to evaluate the erosive potential of commercially available cannabis beverages by first analyzing their pH and fluoride content to characterize their physicochemical properties. Subsequently, the alteration of microhardness and surface roughness of synthetic enamel‐like substrates was assessed before and after exposure. This approach provides essential insight into the potential implications of these products for oral health.

## 2 Materials and Methods

### 2.1 Study Design

This in vitro experimental study was conducted to evaluate the erosive potential of commercially available cannabis beverages. Sixteen test beverages and distilled water as a control were included. The pH and fluoride concentration of each beverage were initially analyzed. Hydroxyapatite blocks were used as enamel substitutes and immersed in each beverage. The alteration of microhardness and surface roughness was measured before and after exposure to assess the erosive effects.

### 2.2 CBD Beverages Selection

The study was waived from ethical approval, as all methodologies did not involve human tissues. The composition and sample coding of each tested beverage are presented in Table 1.

**Table 1:** Samples of tested beverages used in the study.

Samples	Tested beverages	Composition
A	Magic Farm Fresh: Fruit Juice with Cannabis water (Blue Hawaii Flavor)	Cannabis leaf water 50% (from dried cannabis leaf 0.01%), white grape juice (from concentrated white grape juice) 15%, blue hawaii juice 5%, fructose syrup 3%, sugar 2%, stabilizer (INS 466), acidity regulators (INS 330, INS 296), preservative (INS 211), sweeteners (erythritol, sucralose, acesulfame K), artificial color (Allura Red AC, INS 129), artificial flavor.
B	Magic Farm Fresh: Fruit Juice with Cannabis water (Lime Flavor)	Cannabis leaf water 50% (from dried cannabis leaf 0.01%), white grape juice (from concentrated white grape juice) 15%, lime juice (from concentrated lime juice) 5%, fructose syrup 3%, sugar 2%, stabilizer (INS 466), acidity regulators (INS 330, INS 296), preservative (INS 211), sweeteners (erythritol, sucralose, acesulfame K), artificial color (tartrazine, INS 102), artificial flavor.
C	Magic Farm Fresh: Fruit Juice with Cannabis water (Blue pea water lime Flavor)	Cannabis leaf water 50% (from dried cannabis leaf 0.01%), white grape juice (from concentrated white grape juice) 15%, lime juice (from concentrated lime juice) 5%, fructose syrup 3%, sugar 2%, stabilizer (INS 466), acidity regulators (INS 330, INS 296), preservative (INS 211), sweeteners (erythritol, sucralose, acesulfame K), artificial color (tartrazine, INS 102), artificial flavor.
D	Magic Farm Fresh: Fruit Juice with Cannabis water (Mixed berry Flavor)	White grape juice from concentrate 15%, mulberry juice from concentrate 5%, glucose syrup 31%, sugar 28%, Nata de coco 5%, citric acid (INS 330) 0.30%, purple carrot extract (INS 163) 0.25%, nature identical flavoring substances 0.07%, sweetener (sucralose, INS 955) 0.02%, preservative (sodium benzoate, INS 211).
E	Magic Farm Fresh: Fruit Juice with Cannabis water (Lychee Flavor)	Cannabis leaf water 50% (from dried cannabis leaf 0.01%), white grape juice (from concentrated white grape juice) 15%, lychee juice (from concentrated lychee juice) 5%, fructose syrup 3%, sugar 2%, stabilizer (INS 466), acidity regulators (INS 330, INS 296), preservative (INS 211), sweeteners (erythritol, sucralose, acesulfame K), artificial color (Allura Red AC, INS 129), artificial flavor.
F	Magic Farm Fresh: Fruit Juice with Cannabis water (Yuzu Flavor)	Cannabis leaf water 50% (from dried cannabis leaf 0.01%), white grape juice (from concentrated white grape juice) 15%, yuzu juice (from concentrated yuzu juice) 5%, fructose syrup 3%, sugar 2%, stabilizer (INS 466), acidity regulators (INS 330, INS 296), preservative (INS 211), sweeteners (erythritol, sucralose, acesulfame K), artificial color (Allura Red AC, INS 129), artificial flavor.
G	CAMU C Plus	White grape juice from concentrates 10%, sugar 0.6%, vitamin C 0.080%, hemp leaf powder 0.01%, GABA 0.006%, Camu camu powder 0.025%, vitamin B12 0.00015%, stabilizers (INS 440, INS 418), acidity regulators (INS 330, INS 296, INS 331(iii)), sweeteners (sucralose, maltitol syrup), natural color (INS 141(ii)), nature‐identical flavoring.
H	Tipco Leafly Cannabis water (Original)	Cannabis leaf infusion (from real cannabis leaves), 0% sugar, 0% calories, natural herbal aroma.
I	Tipco Leafly Cannabis water (Yusu Orange)	Cannabis leaf infusion (from real cannabis leaves), yuzu orange flavor, sugar, 0% calories, natural herbal aroma.
J	Cannabis water (Yanhee Lavender Mixed Berries)	Cannabis leaf water 99.88%, vitamin B12 0.00000022%, food additives (INS 296), sweeteners (erythritol, sucralose).
K	DEK420: Cannabis Drink (Original)	Cannabis leaves 3%, acidity regulator (INS: 575), preservative (INS:211, INS:202), artificial flavor.
L	DEK420: Cannabis Drink (Pandan)	Cannabis water 60% (cannabis Leaves 3%), pandan water 35.9%, sugar 4%, natural flavor, preservative (INS:211), acidity regulator (INS: 575), artificial color, sweetener (steviol glycosides).
M	DEK420: Cannabis Drink (Chrysanthemum)	Cannabis water 60% (cannabis leaves 3%), chrysanthemum water 35.87%, sugar 4%, natural flavor, acidity regulator (INS: 575), preservative (INS:211), artificial color (INS:102), artificial flavor, sweetener (steviol glycosides).
N	DEK420: Cannabis Drink (Honey Lemon Flavor)	Cannabis water 95.79% (cannabis leaves 3%), sugar 4%, acidity regulator (INS:330, INS: 575), natural flavor, preservative (INS:211), artificial flavor, sweetener (steviol glycosides).
O	DEK420: Cannabis Drink (Roselle)	Cannabis water 60% (cannabis leaves 3%), roselle juice 35.81%, sugar 4%, acidity regulator (INS:330), natural flavor, preservative (INS:211), artificial flavor, sweetener (steviol glycosides).
P	DEK420: Cannabis Drink (Blue Hawaii)	Cannabis water 95.36% (cannabis leaves 3%), sugar 4%, salt 0.02%, acidity regulator (INS:330), natural flavor, natural identical flavor, artificial flavor, preservative (INS:211), artificial color (INS:331), sweetener (steviol glycosides).
Control	Distilled water, Dental Materials Research and Development Center	Distilled water

### 2.3 Fluoride Concentration and pH Measurement

Sixteen commercially available beverages were freshly prepared in 5 mL without dilution. Fluoride concentration was measured using a fluoride ion‐selective electrode (Versa Star; Thermo Fisher Scientific Inc., Waltham, MA, USA), calibrated with external fluoride standards at 1000, 100, 10, and 1 ppm. Before the measurement, each sample was mixed with Total Ionic Strength Adjustment Buffer (TISAB IV) and 70% perchloric acid (Merck, Darmstadt, Germany) at a 1:10 ratio to ensure appropriate ionic strength and pH buffering for accurate fluoride detection, and the mean fluoride concentration (ppm F) was calculated. The pH of the beverages was measured using a calibrated pH meter (SevenEasy S20; Mettler‐Toledo, Switzerland), with standard buffer solutions at pH 4.01, 7.00, and 10.01 (Mettler‐Toledo, Switzerland). All fluoride and pH measurements were performed in triplicate, with electrodes rinsed with distilled water and dried between readings to prevent cross‐contamination.

### 2.4 Sample Size Calculation and Sample Preparation

The sample size was determined using G*Power software (version 3.1), based on parameters derived from previous in vitro studies by Lussi et al. on the erosive effects of different soft drinks on enamel surfaces (Lussi et al. 2023), with an effect size of 0.90, *α* = 0.05, and a statistical power of 80%, resulting in a minimum requirement of five specimens per group.

Synthetic hydroxyapatite blocks (2500 psi; NSTDA, Bangkok, Thailand) served as enamel substitutes. This material has a chemical composition similar to that of natural enamel, primarily consisting of calcium phosphate (Ca_10_(PO_4_)_6_(OH)_2_), and previous studies (Rujiraprasert et al. 2023) have confirmed that its mechanical properties closely resemble those of enamel, providing a reliable alternative due to infection control considerations.

Eighty‐five hydroxyapatite blocks were fabricated in a cylindrical form (5 mm diameter × 5 mm thickness) and allocated into 16 experimental groups (tested beverages) and 1 control group (distilled water). The blocks were embedded in epoxy resin molds using commercial epoxy resin (Bangkok, Thailand) and allowed to set for 24 h. After complete curing, the specimens were wet‐polished using a polishing machine (NANO2000, PACE Technologies, Arizona, USA) at 300 rpm for 15 s to obtain a smooth and standardized surface. Polishing was performed sequentially with 600‐, 800‐, 1200‐, and 2400‐grit silicon carbide papers (TOA, Bangkok, Thailand), followed by final polishing with alumina oxide powder. The specimens were then ultrasonically cleaned in distilled water. After cleaning, each specimen was examined under a 10× stereomicroscope (SZ61, Olympus, Japan), and any specimen exhibiting surface defects was excluded. The polished surface of each specimen was divided into two regions: the left side for baseline measurements and the right side for post‐immersion evaluation.

### 2.5 Microhardness and Surface Roughness Test

Knoop microhardness was measured using a microhardness tester (FM810, Future‐Tech Corp., Kanagawa, Japan) with a 100 g load and a dwell time of 15 s. Indentations were initiated 100 µm from the margin and spaced at 100 µm intervals. Five indentations were performed on the left side of each specimen for baseline measurement, and the Knoop Hardness Number (KHN) values were recorded to obtain the mean. Following the immersion procedure, Knoop microhardness was re‐evaluated on the right side of each specimen using the same protocol. Five indentations were performed for post‐immersion measurement, and the mean and standard deviation were calculated for statistical analysis. Moreover, the percentage of surface hardness loss (%SHL) was calculated using the following formula: %SHL = [(initial microhardness − final microhardness)/initial microhardness] × 100, as previously described (Paiva et al. 2017; Martins et al. 2025).

Surface roughness was measured using a contact‐type profilometer (Talyscan 150, Taylor Hobson Ltd., Leicester, England) equipped with a 5‐µm diamond stylus tip. Each specimen was secured on a fixation stand, and the stylus was positioned to scan a 0.5 × 0.5 mm^2^ area corresponding to the region used for microhardness testing. A constant load of 5 N was applied at a scanning speed of 1500 µm/s. Five parallel traces were recorded on the left side of each specimen for baseline measurement at intervals of 100 µm, and the mean surface roughness (Ra) value was calculated. Following the immersion procedure, surface roughness was re‐evaluated on the right side of each specimen using the same protocol. Five parallel traces were recorded for post‐immersion measurement, and the mean and standard deviation were calculated for statistical analysis. The percentage change in surface roughness (%Raalt) was calculated using the following formula: %Raalt = [(final surface roughness − initial surface roughness)/initial surface roughness] × 100, according to previously described methods (da Silva et al. 2024).

### 2.6 Sample Immersion Procedure

All test beverages, including distilled water as the control, were freshly prepared prior to the experiment. Specimens in both experimental and control groups were individually immersed in 5 mL of the assigned test beverage for 30 min under controlled temperature conditions at 37°C (LK LABKOREA, LI‐IS450, Korea). Following immersion, the specimens were rinsed with distilled water using an ultrasonic cleaner (GT SONIC, VGT‐1990 QTD, China) for 10 s and subsequently dried at 37°C with blue silica gel (TOA, Bangkok, Thailand) for 1 h prior to post‐immersion microhardness and surface roughness measurements.

### 2.7 Statistical Analyses

All data were expressed as mean ± standard deviation. Statistical analyses were performed using GraphPad Prism version 9.1.1 (GraphPad Software, San Diego, CA, USA). Data normality for microhardness, %SHL, surface roughness, %Raalt, fluoride concentration, and pH were assessed using the Shapiro–Wilk test, and homogeneity of variance was evaluated using Levene's test. All data sets showed a normal distribution (*p* > 0.05); therefore, parametric statistical analyses were considered appropriate. Differences among groups were analyzed using one‐way analysis of variance (ANOVA), followed by Tukey's post hoc test for multiple comparisons. A *p* < 0.05 was considered statistically significant.

## 3 Results

Table 2 presented the fluoride concentration (ppm), pH values, %SHL, and %Raalt, expressed as mean ± standard deviation. Statistically significant differences among groups were indicated.

**Table 2:** Fluoride concentration, pH, %SHL, and %Raalt of cannabis beverages (mean ± SD).

Samples	Fluoride concentration mean (SD)	pH mean (SD)	%SHL (SD)	%Raalt (SD)
Sample A	0.54 (0.009)^F^	3.41 (0.006)^b^	31.01% (5.01)^γ^	22.81% (1.91)^‡^
Sample B	0.55 (0.059)^F^	3.17 (0.015)^c^	27.52% (4.41)^γ^	11.41% (0.54)^†^
Sample C	0.53 (0.004)^F^	3.19 (0.012)^c^	53.14% (6.87)^ζ^	214.66% (5.92)^¶^
Sample D	0.55 (0.008)^F^	3.26 (0.006)^c^	57.66% (7.35)^ζ^	55.74% (2.26)^§^
Sample E	0.56 (0.005)^F^	3.37 (0.006)^b^	46.45% (3.52)^ε^	46.62% (1.76)^§^
Sample F	0.53 (0.006)^F^	3.17 (0.026)^c^	41.67% (1.54)^ε^	106.97% (3.23)^¶^
Sample G	0.15 (0.009)^E^	3.49 (0.006)^b^	35.46% (9.45)^δ^	21.84% (9.45)^‡^
Sample H	0.06 (0.030)^B,C^	7.18 (0.240)^a^	3.80% (0.94)^α^	45.80% (2.88)^§^
Sample I	0.04 (0.003)^A,B^	5.97 (1.724)^a^	6.95% (0.40)^α^	29.17% (5.98)^‡^
Sample J	1.82 (0.006)^G^	4.30 (0.012)^b^	11.77% (4.49)^β^	43.40% (8.70)^§^
Sample K	0.14 (0.004)^D,E^	4.52 (0.006)^b^	−15.50% (7.93)^α^	7.15% (1.44)^†^
Sample L	0.07 (0.002)^B,C^	4.01 (0.010)^b^	26.34% (7.32)^γ^	16.45% (1.33)^‡^
Sample M	0.03 (0.003)^A,B^	3.99 (0.006)^b^	15.95% (4.01)^β^	5.23 (1.12)^†^
Sample N	0.07 (0.000)^B,C^	2.84 (0.006)^d^	73.78% (7.42)^η^	733.49% (6.68)^★^
Sample O	0.09 (0.005)^C,D^	2.66 (0.020)^e^	73.15% (5.31)^η^	982.81% (13.86)^★★^
Sample P	0.03 (0.003)^A,B^	2.84 (0.000)^d^	56.46% (4.43)^ζ^	408.43% (22.94)^♦^
Control	0.00 (0.000)^A^	7.00 (0.000)^a^	0.20% (2.12)^α^	0.50% (0.30)^†^

*Note:* Uppercase letters indicated statistically significant differences among groups for fluoride concentration, whereas lowercase letters indicated statistically significant differences for pH. Greek letters (α–η) indicated statistically significant differences for %SHL, and symbols (†, ‡, §, ¶, ★, etc.) indicated statistically significant differences for %Raalt (*p* < 0.05). Values sharing the same letter or symbol were not significantly different.

### 3.1 Fluoride Concentration and pH Measurement

Fluoride concentrations across the beverages ranged from 0.03 ± 0.003 mg/L to 1.82 ± 0.006 mg/L, indicating generally low fluoride content. Most cannabis beverages exhibited highly acidic conditions, with samples A, B, C, D, E, F, G, M, N, O, and P showing pH values below 4. Sample H was the only product with a neutral pH (7.18), while sample I showed a mildly acidic pH of 5.97. The control sample exhibited a neutral pH of 7.00.

### 3.2 Microhardness Test

The microhardness results, expressed as %SHL, were presented in Table 2. Significant differences in %SHL were observed among the tested beverages (*p* < 0.05). The highest %SHL values were associated with beverages of lower pH, particularly samples N, O, and P. In contrast, samples H and I exhibited minimal %SHL values comparable to the control group. Sample K showed a negative %SHL value, suggesting a potential surface hardening effect.

### 3.3 Surface Roughness Test

The surface roughness results, expressed as %Raalt, are presented in Table 2. Significant differences in %Raalt were observed among the tested beverages (*p* < 0.05). The highest %Raalt values were associated with beverages of lower pH, particularly samples N, O, and P. In contrast, samples with higher pH exhibited lower %Raalt values comparable to the control group.

## 4 Discussion

The erosive potential of commercially available cannabis beverages was a primary focus of this study, and the findings demonstrated that several products produced measurable erosive effects on enamel‐like surfaces. Hydroxyapatite blocks were selected as the enamel substitute because this material, synthesized from calcium phosphate‐based compounds, following established protocols (Rujiraprasert et al. 2023), had already been proven to serve as a standardized model for enamel substrate while avoiding the ethical and biosafety concerns associated with using extracted human teeth.

CBD beverages included in this experiment contained acidic additives, fruit concentrates, sweeteners, preservatives, and cannabis‐derived components. Such ingredients—particularly citric acid, phosphoric acid, and low pH—can promote enamel demineralization by dissolving hydroxyapatite and softening the tooth surface (Lussi et al. 2012). The critical pH of enamel, at which demineralization begins, is approximately 5.5 (Meurman and ten Gate 1996), a level comparable to the behavior of hydroxyapatite. The results of the erosive potential, assessed through microhardness and surface roughness testing, revealed a marked reduction in hardness among beverages with pH values below 4. This finding aligns with previous evidence, demonstrating that acidic beverages rapidly initiate surface demineralization (Barac et al. 2015; Maladkar et al. 2022). When an acidic solution contacts the surface, hydrogen ions diffuse into the hydroxyapatite lattice and replace calcium and phosphate ions, leading to dissolution of mineral content and progressive softening of the substrate (Harper et al. 2021). Additionally, repeated or prolonged exposure further enhances mineral loss because the low‐pH environment disrupts the natural balance between remineralization and demineralization, resulting in surface erosion (Abou Neel et al. 2016). However, not all cannabis beverages exhibited the same level of erosive potential. Beverages with a pH closer to neutral, such as sample H (pH 7.18) and Sample I (pH 5.97), did not significantly reduce the microhardness of the hydroxyapatite, which aligns with the characteristics of less erosive or nonerosive beverages. Notably, the %SHL values of these samples were also comparable to the negative control, further supporting their minimal erosive potential. However, despite this favorable performance in surface hardness, these samples still exhibited moderate %Raalt values compared with other groups, suggesting that surface alterations may occur even in the absence of substantial mineral loss. These findings indicate that pH plays a critical role in determining the erosive potential of cannabis beverages, consistent with observations reported for other acidic drinks (Inchingolo et al. 2023).

This study also assessed the fluoride content of cannabis beverages, as previous research had shown that some herbal drinks may contain elevated fluoride levels (Szmagara et al. 2022). Fluoride in beverages can influence oral health by promoting remineralization of tooth structure and providing certain antimicrobial benefits. However, the low fluoride concentrations detected in most samples in this study suggest that these protective effects are unlikely to be clinically meaningful, as observed reductions in microhardness and increases in surface roughness. An exception was cannabis beverage Sample J, which contained 1.8233 ± 0.006 mg/L of fluoride. This level exceeded established safety guidelines. The World Health Organization recommends that fluoride concentrations in drinking water in tropical regions should not exceed 0.8 mg/L due to higher daily water intake, while Thailand sets an even lower guideline of 0.7 mg/L, based on an estimated consumption of 2 L of water per day for a 60‐kg adult (Sawangjang et al. 2019; DoH, Ministry of Public Health 2010). Therefore, the fluoride content of Sample J exceeded both the WHO and Thai recommendations for a safe daily intake, underscoring the need for careful monitoring of fluoride levels in these beverage products.

The increase in surface roughness highlights another important aspect of dental erosion. Surface irregularities may facilitate greater plaque accumulation, which can contribute to an increased risk of demineralization and dental caries when combined with the presence of fermentable carbohydrates (Serbanoiu et al. 2024). In the present study, alterations in the hydroxyapatite surfaces were also associated with the pH levels. Based on previous research, acids from beverages can affect tooth enamel for approximately 20–30 min following consumption, during which demineralization predominates before salivary buffering occurs (Abou Neel et al. 2016). When beverages are consumed slowly over an extended period, this acidic challenge may be prolonged. Therefore, a 30‐min immersion period was selected to represent a sustained erosive exposure and to enable standardized comparison among the tested beverages.

A 30‐min immersion in low‐pH samples produced notably greater increases in surface roughness than beverages with higher pH values. If such beverages are consumed frequently without proper preventive measures, the erosive effects may accumulate over time. This interpretation is supported by previous research demonstrating that the severity of erosion increases in proportion to exposure duration (Barac et al. 2015), reinforcing the critical role of acidic conditions in promoting mineral loss. However, the finding that not all cannabis beverages induced erosion suggested that the erosive potential was influenced primarily by the overall beverage formulation—such as acidity, buffering capacity, and added ingredients—rather than by the cannabis extract itself.

The results of this study showed that the %SHL and the %Raalt in surface roughness followed a consistent trend and were closely associated with beverage pH. Specifically, lower pH values were correlated with higher levels of demineralization and greater increases in surface roughness, indicating that acidity plays a critical role in mineral loss and surface alteration. These findings suggest that highly acidic beverages accelerate the dissolution of hydroxyapatite, leading to structural weakening and increased surface irregularity. Interestingly, sample K showed a negative %SHL value together with the lowest %Raalt, indicating apparent surface hardening with minimal surface alteration. This may be related to its formulation and relatively higher pH, which could limit hydroxyapatite dissolution or promote surface deposition. However, as mineral gain was not directly assessed, this finding should not be interpreted as definitive remineralization but rather as a possible surface hardening or precipitation effect (Shellis et al. 2011).

Additionally, it is important to note that the conditions of direct exposure in these in vitro microhardness and surface roughness assessments may not fully represent real‐life consumption. In the oral environment, saliva plays a protective role through buffering capacity, salivary clearance, and pellicle formation, all of which can reduce acid contact and promote remineralization (Baumann et al. 2016; C. Hannig and M. Hannig 2025). Erosive tooth wear is defined as the cumulative loss of mineralized tooth structure, resulting from chemical and mechanical processes, in which dental erosion—defined as the chemical dissolution of tooth structure by acids not derived from oral bacteria—acts as the primary etiological factor. This multifactorial process involves interactions between chemical erosion and mechanical forces such as mastication and toothbrushing, which may further accelerate surface loss (Schlueter et al. 2020). Therefore, the extent of surface alteration observed under experimental conditions may differ from that in clinical settings.

A limitation of this study is that each beverage contained multiple additional ingredients, and neither a cannabinoid‐free control beverage nor a pure cannabinoid formulation was included, as such products are not readily available on the commercial market and do not represent commonly consumed ready‐to‐drink beverages. These additional components may have influenced the observed effects, which may therefore reflect combined interactions among formulation ingredients rather than cannabis‐derived constituents alone. Within these limitations, the present study provides important insight into the erosive potential of commercially available cannabis beverages and highlights the critical role of physicochemical properties, particularly acidity, in influencing the integrity of synthetic enamel‐like hydroxyapatite substrates.

## 5 Conclusion

This study demonstrated that several commercially available cannabis beverages reduced microhardness and increased surface roughness of hydroxyapatite blocks, particularly those with pH values below 4. These findings indicate that beverage acidity plays an important role in enamel demineralization, suggesting that the physicochemical properties of cannabis beverages, especially acidity, should be considered when evaluating their potential effects on dental hard tissues.

## Author Contributions

Boonprakong Boonsuth, Ajima Chansaenroj, and Thawanrat Singthong contributed to data curation and data analysis. Sureeporn Tantisak contributed to data analysis. Dusit Nantanapiboon and Supreda Suphanantachat Srithanyarat contributed to the experimental design and data analysis, with Dusit Nantanapiboon also responsible for funding acquisition and final manuscript preparation. Vorapat Trachoo, Sant Ananchanachai, and Naruporn Monmaturapoj contributed to resources and data interpretation. Junji Tagami contributed to data interpretation. Thanaphum Osathanon contributed to conceptual design, supervision, and manuscript drafting. All authors critically reviewed the manuscript and approved the final version for publication.

## Ethics Statement

The study was waived from ethical approval, as all methodologies did not involve human tissues.

## Conflicts of Interest

The authors declare no conflicts of interest.

## Data Availability

The data that support the findings of this study are available from the corresponding author upon reasonable request.
